# Atrial electrophysiological and molecular remodelling induced by obstructive sleep apnoea

**DOI:** 10.1111/jcmm.13145

**Published:** 2017-04-12

**Authors:** Devika Channaveerappa, Jacob C. Lux, Kelly L. Wormwood, Timothy A. Heintz, Meredith McLerie, Jacqueline A. Treat, Hannah King, Donia Alnasser, Robert J. Goodrow, Glenn Ballard, Robert Decker, Costel C. Darie, Brian K. Panama

**Affiliations:** ^1^ Biochemistry and Proteomics Group Department of Chemistry and Biomolecular Science Clarkson University Potsdam NY USA; ^2^ Department of Experimental Cardiology Masonic Medical Research Laboratory Utica NY USA; ^3^ Electrical Engineering Technology Mohawk Valley Community College Utica NY USA

**Keywords:** obstructive sleep apnoea, atria, protein, ECG, proteomics

## Abstract

Obstructive sleep apnoea (OSA) affects 9–24% of the adult population. OSA is associated with atrial disease, including atrial enlargement, fibrosis and arrhythmias. Despite the link between OSA and cardiac disease, the molecular changes in the heart which occur with OSA remain elusive. To study OSA‐induced cardiac changes, we utilized a recently developed rat model which closely recapitulates the characteristics of OSA. Male Sprague Dawley rats, aged 50–70 days, received surgically implanted tracheal balloons which were inflated to cause transient airway obstructions. Rats were given 60 apnoeas per hour of either 13 sec. (moderate apnoea) or 23 sec. (severe apnoea), 8 hrs per day for 2 weeks. Controls received implants, but no inflations were made. Pulse oximetry measurements were taken at regular intervals, and post‐apnoea ECGs were recorded. Rats had longer P wave durations and increased T wave amplitudes following chronic OSA. Proteomic analysis of the atrial tissue homogenates revealed that three of the nine enzymes in glycolysis, and two proteins related to oxidative phosphorylation, were down regulated in the severe apnoea group. Several sarcomeric and pro‐hypertrophic proteins were also up regulated with OSA. Chronic OSA causes proteins changes in the atria which suggest impairment of energy metabolism and enhancement of hypertrophy.

## Introduction

Obstructive sleep apnoea is characterized by transient cessations in respiration lasting more than 10 sec. as a result of narrowing or occlusion of the upper airway during sleep 1. OSA severity is assessed by the apnoea‐hypopnea index (AHI), or the average number of apnoeas (complete obstructions) or hypopneas (partial obstructions) per hour 2. An estimated 20% of adults have mild OSA (AHI 5–15) and 7% of adults have moderate (AHI 15‐30) to severe (AHI > 30) OSA, with 85% of all patients remaining undiagnosed 1, 2. Obstructive apnoea causes transient hypoxia as well as decreased intrathoracic pressure because of enhanced breathing effort on the collapsed airway 2, 3. OSA is associated with daytime sleepiness, headache, depression, hypertension, obesity, arthritis, type 2 diabetes and heart disease 1, 4, 5. There has been interest in the link between OSA and atrial pathologies as OSA patients have an increased incidence of atrial arrhythmias and left atrial enlargement 6. Although clinical evidence linking OSA to atrial pathology is known, the molecular mechanisms by which OSA causes atrial disease remain elusive. Furthermore, most animal models do not accurately recapitulate OSA as it occurs in humans. For example, many studies utilize chronic intermittent hypoxia as a model of OSA 7. This model induces transient hypoxia, but it does not produce the upper airway obstructions observed in clinical cases of OSA and, consequently, does not cause characteristic changes in intrathoracic pressure. While other models have reproduced airway obstructions 8, 9, these animals need to be anesthetized throughout the experiment.

To obviously define the molecular changes in atria which are caused by OSA, we have implemented a recently developed OSA model, involving a tracheal balloon as the obstruction device which produces apnoeas in conscious and free‐roaming rats 10, 11. This model consistently recapitulates the features of OSA. We found that chronic OSA for 2 weeks was associated with increases in both P wave duration and T wave amplitude. To further investigate cardiac changes caused by OSA, we examined the proteomes of the atria from rats with 2 weeks of chronic apnoea. Significant dysregulations were observed for metabolic proteins, suggesting a decrease in glycolysis and a diminished ability to produce reducing equivalents for ATP generation. Structural and sarcomere proteins were also up regulated, which is consistent with cardiac hypertrophy. These results show the complex pattern of changes that occurs with OSA and contributes to the development of cardiac disease.

## Materials and methods

### OSA model

**Figure 1:**
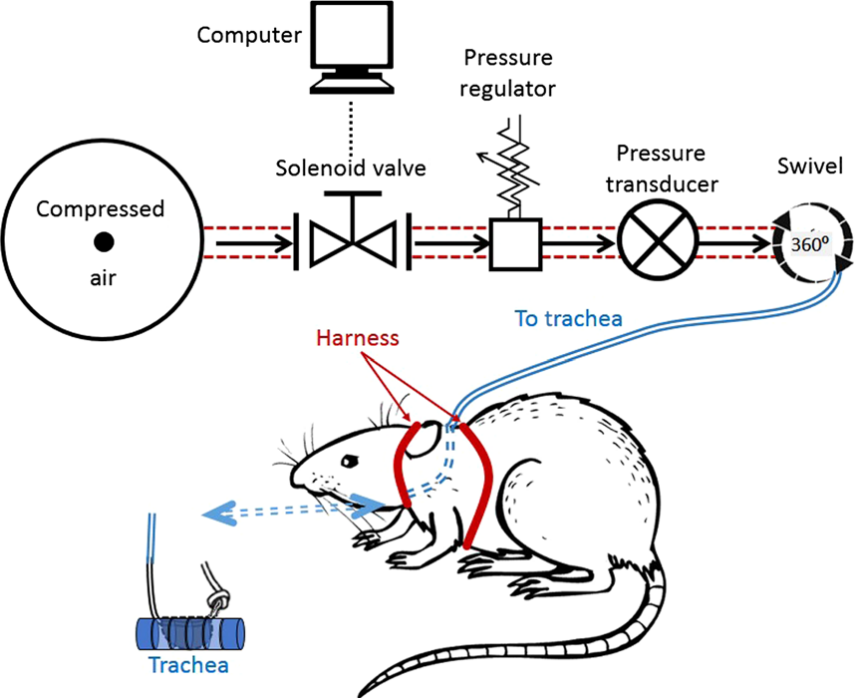
A schematic diagram of the obstructive sleep apnoea setup. To produce an apnoea, a surgically implanted balloon is inflated in the trachea of a rat. Rats are free ranging and allowed access to food/water. The tracheal balloon is inflated by an automated pressure system, where rats were give 13 or 23 sec. apnoeas, 60 times per hour for 8 hrs.

The use of male Sprague Dawley rats (aged 50–70 days) conformed to the Guide for the Care and Use of Laboratory Animals, published by the National Institutes of Health (Eighth Edition, 2011). All research protocols were approved by the Animal Care and Use Committee at the Masonic Medical Research Laboratory. Rats were housed in individual cages for the duration of the experiment and were subjected to a 12‐hrs light–dark cycle. The apnoea surgery was similar to that described in Crossland *et al*. 10. Tracheal obstructive apnoea devices were made from 3 cm of silicone tubing (RenaSil .037″ OD .025″ ID, SIL 037; Braintree Scientific, Braintree, MA, USA). Silicone tubing was tied on one end with an overhand knot and stretched 5–10 times. The tubing was rotated 90° longitudinally and stretched 5–10 additional times to produce a balloon that measuring 3 mm in width and 4 mm in length when inflated. Silicone tubing was affixed to an 11 cm length of PE50 tubing (PE50; Braintree Scientific) using two 4‐0 sutures tied around the junction. The connection was coated with nail polish, dried and wrapped with parafilm. Initial inflation pressures measured between 17 and 25 psi. Rats were anesthetized with isoflurane (5% for induction and 1.5–2% for maintenance). A 2.5 cm midline incision was made in the ventral portion of the neck. The trachea was isolated using blunt dissection, and two holes were made in the trachea. The first hole was two tracheal rings below the larynx, and the second hole was three tracheal rings (3 mm) distal to the first. The obstruction device was threaded through tracheal holes and secured in place with two 6‐0 sutures. A 5 mm midline incision was made dorsally between the scapulae, and the PE50 tubing end of the device was tunnelled to the dorsal incision subdermally and fixed to a 23‐gauge stainless steel L‐shaped tube (3 cm) using cyanoacrylate and silicone tubing (ID .058″ OD .077″ SIL‐6‐25; SAI, Lake Villa, IL, USA). The sternohyoid muscle and dermis were closed with sutures. Triple antibiotic (Actavis, Parsippany, NJ, USA) was applied topically to the wound sites, and rats were administered buprenorphine (30 μg/kg) for pain immediately following the surgery. Rats were free ranging and allowed full access to food and water, while they were connected to the apnoea system. The PE50 tubing was threaded through a 16″ or 18″ harness‐swivel configuration (SAH‐18; SAI) (see Fig. 1). Clavamox (40 mg/kg) (Dr. Fosters and Smith, Co., Rhinelander, WI, USA) was given PO on a five‐day regimen, beginning one day before surgery. Following one week of recovery, apnoeas lasting 13 or 23 sec. were randomly delivered via computer‐controlled compressed air system (Fig. 1). Apnoeas were confirmed observing O_2_ desaturation in conjunction with increased breathing effort upon balloon inflation. Control rats received implants, but no inflations were made.

### Pulse oximetry

Pulse oximetry was performed at five‐day intervals on conscious rats using the Mouse Ox pulse oximetry system (Starr Life Sciences, Oakmont, PA, USA). Baseline O₂ was established in all rats, and apnoeas were evaluated for consistency and maximum desaturation. In the 13 sec. group, fluctuations in the nadir O₂ saturation occurred over the 14‐day apnoea period (*P* < 0.05), but there was no trend. The 23 sec. group displayed no variation in O_2_ nadir throughout the experiment (*P* = 0.13).

### Electrocardiogram recording

Electrocardiograms (Lead II) were recorded using subcutaneous electrodes. ECG recordings were performed using an Iso‐DAM8A amplifier (World Precision Instruments, Sarasota, FL, USA). Signals were digitized with CED Power 1401 digital converter and acquired with Spike 2 software (Cambridge Electronic Design Limited, Cambridge, UK). Rats were anesthetized using 1.5–2% isoflurane, and the temperature was maintained at 35–36°C. Approximately 400 sec. of stable, continuous ECG data were analysed in Labchart 7 Pro (ECG Module) (ADInstruments, Colorado Springs, CO, USA). The QTc was calculated using Bazett's formula, and ‘QTcr’ represents the correction from Kmecova *et al*. 12: $\left(\mathrm{QTcr}=\mathrm{QT}/\sqrt{\mathrm{RR}/0.15}\right)$. Beats with breathing artefacts or isoelectric line noise fluctuations were eliminated from the data.

### Proteomic analysis

Rats were anesthetized by an IP injection of ketamine (80 mg/kg)/xylazine (8 mg/kg). Hearts were rapidly removed, washed in chilled Tyrode's solution and flash frozen with liquid N_2_. Atrial tissue was disrupted in liquid nitrogen with a mortar and pestle. The disrupted tissue was placed in 300 μl of chilled lysis solution containing NaCl 100 mM, Tris‐HCl 25 mM, EDTA 0.2 mM, NaF 2 mM, Na_3_VO_4_ 2 mM and Complete Protease Inhibitor (Sigma, St. Louis, MO, USA). The tissue was homogenized in the solution by repeatedly passing the solution through sterile 18‐ and 22‐gauge needles. The homogenate was centrifuged at 16,000 × g for 5 min., and the supernatant was collected and placed on ice. The pellet was resolubilized in 300 μl of lysis buffer containing 2% NP40. After incubation on ice for 1 hr, the sample was again centrifuged at 16,000 × g for 5 min. and the supernatant was collected and set aside on ice. The pellet then was solubilized a third time in 200 μl of lysis buffer containing 4% SDS and centrifuged at 16,000 × g for 5 min. The supernatant was removed and combined with the two previous supernatants. The protein was quantified using BCA Assay (ThermoFisher Scientific, Waltham, MA, USA), and samples were kept at −80°C until use.

Both the supernatants and the pellets were separated on a large format, home‐made 12% SDS‐PAGE, as described in 13. For proteomics analysis, only the supernatants were used. Exactly 20 μg of protein from each sample was loaded on to the gel. Once the gels were run, they were stained by Coomassie, and the gel lanes for control, moderate and severe apnoea were divided into gel pieces and then subjected to trypsin in‐gel digestion, as described previously 14. The resulting peptides were then extracted, dried in a Speedvac and then cleaned with a C18 Ziptip and further solubilized in 2% (v/v) acetonitrile (ACN)/0.1% (v/v) formic acid (FA) in HPLC water.

The peptides mixture was analysed by reversed phase liquid chromatography (LC) and MS (LC‐MS/MS) using a NanoAcquity UPLC (Waters, Milford, MA, USA) coupled to a Q‐TOF Xevo G2 MS (Waters), according to published procedures 13, 15, 16, 17. Briefly, the peptides were loaded onto a 100 μm × 10 mm NanoAquity BEH130 C18 1.7 μm UPLC column (Waters) and eluted over a 180‐min. gradient at a flow rate of 400 nL/min as follows: 2–45% organic solvent B (ACN containing 0.1% FA) over 1–120 min., 45–85% B (120–140 min.), constant 85% B (140–160 min.), 85%‐2% B (160–165 min.) and then return to the initial conditions of 2% B (165–180 min.). The aqueous solvent A was 0.1% FA in HPLC water. The column was coupled to a Picotip Emitter Silicatip nano‐electrospray needle (New Objective, Woburn, MA, USA). MS data acquisition involved survey 0.5 sec. MS scans (m/z range 350–2000) and automatic data dependent analysis (DDA) of the top six ions with the highest intensity, with the charge of 2+ , 3+  or 4+ . The MS/MS (recorded over m/z of 50–2000) was triggered when the MS signal intensity exceeded 500 counts/sec. In survey MS scans, the six most intense peaks were selected for collision‐induced dissociation (CID) and fragmented until the total MS/MS ion counts reached 6000 or for up to 1.1 sec. each. The entire procedure used was previously described 13, 15, 16. Calibration was performed for both precursor and product ions using 1 pmol GluFib (Glu1‐Fibrinopeptide B) standard peptide with the sequence EGVNDNEEGFFSAR and the monoisotopic doubly charged peak with m/z of 785.84.

### Data processing and protein identification

The raw data were processed using ProteinLynx Global Server (PLGS, version 2.4 Waters Corporation, Milford, MA) software, as described previously 16. The following parameters were used: background subtraction of polynomial order five adaptive with a threshold of 30%, two smoothings with a window of three channels in Savitzky‐Golay mode and centroid calculation of top 80% of peaks based on a minimum peak width of four channels at half height. The resulting pkl files were submitted for database search and protein identification to the in‐house Mascot server for database search (www.matrixscience.com, Matrix Science, London, UK, version 2.5.1) using the following parameters: databases from NCBI_20150706 database (selected for Rattus, 84,157 entries), parent mass error of 1.3 Da, product ion error of 0.8 Da, one ^13^C isotope, enzyme used: trypsin, one missed cleavage, propionamide as cysteine fixed modification and methionine oxidized as variable modification. To identify the false‐negative results, we used additional parameters such as different databases or organisms, a narrower error window for the parent mass error (1.2 and then 0.2 Da) and for the product ion error (0.6 Da), and up to two missed cleavage sites for trypsin. In addition, the pkl files were also searched against in‐house PLGS database version 2.4 (Waters Corporation, Milford, MA, USA) using searching parameters similar to the ones used for Mascot search. The Mascot and PLGS database search provided a list of proteins for each gel band. These data were then uploaded on the Scaffold version 4.2.1 software (Proteome Software Inc., Portland, OR, USA) for quantitative analysis.

### Criteria for protein identification

Scaffold (version Scaffold_4.2.1; Proteome Software Inc., Portland, OR, USA) was used to validate MS/MS based peptide and protein identifications. Peptide identifications were accepted if they could be established at >20.0% probability by the Peptide Prophet algorithm 18 with Scaffold delta‐mass correction. Protein identifications were accepted if they could be established for correction, and if they could be established at >99.0% probability and contained at least 1 identified peptide. Protein probabilities were assigned by the Protein Prophet algorithm 19. Proteins that contained similar peptides and could not be differentiated based on MS/MS analysis alone were grouped to satisfy the principles of parsimony. Proteins sharing significant peptide evidence were grouped into clusters. Proteins were annotated with GO terms from NCBI (downloaded August 9, 2016) 20.

### Data sharing

Raw data and the Scaffold file, as well as the HTML files of the Mascot database search will be provided upon request, according to Clarkson University's Material Transfer Agreement.

### Statistical analysis

Data are presented as Mean ± S.E.M. Statistical comparisons of two means were made using paired or unpaired Student's *t*‐test where appropriate. A one way ANOVA followed by Bonferroni post‐test was used for multiple comparisons. A *P* < 0.05 was considered statistically significant (*).

## Results

### Cardiac changes in OSA rats

**Figure 2:**
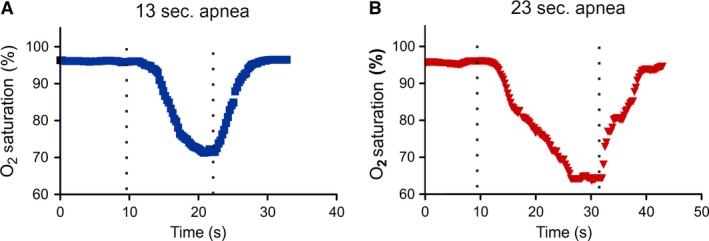
Examples of obstructive sleep apnoea pulse oximetry data O_2_ saturation waveforms during apnoeas. Both 13 sec. (**A**) and 23 sec. (**B**) apnoea caused transient decreases in O_2_ saturation. The apnoea duration is denoted between the broken lines.

**Figure 3:**
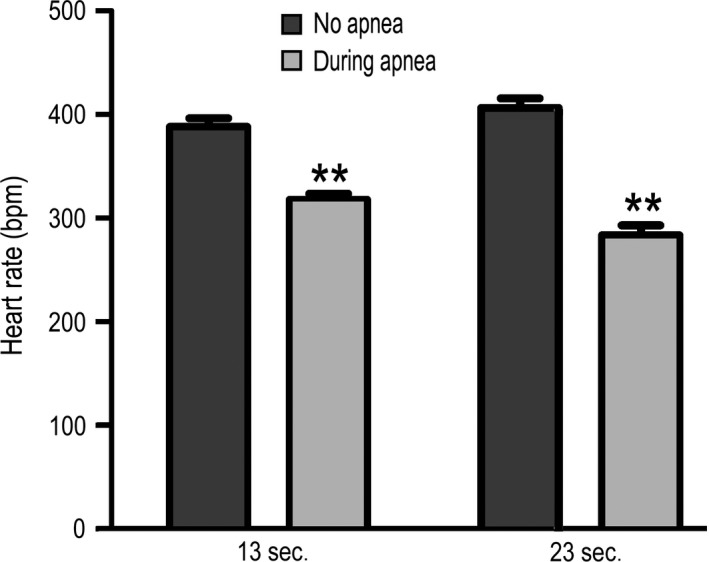
Average normoxic and minimum hypoxic heart rate. 13 and 23 sec. apnoeas resulted in a 70.4 ± 14.7 and 122.5 ± 18.9 beat per minute decrease, respectively (*P* < 0.01).

**Figure 4:**
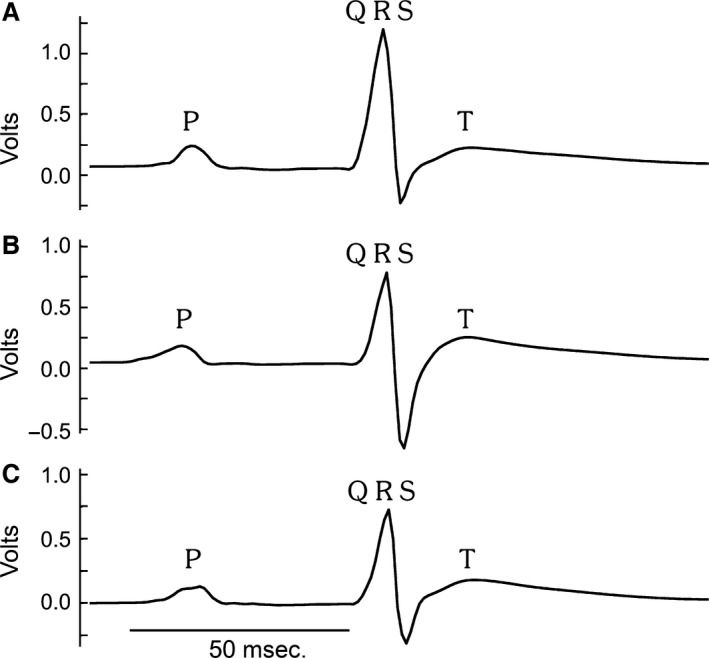
Averaged ECGs. Examples of averaged ECGs from control (**A**) and chronic obstructive sleep apnoea rats, moderate (**B**) and severe (**C**). P, QRS and T waves are labelled.

The apnoea model, as depicted in Figure 1, recapitulated the pathophysiological characteristics of OSA 10, 11. We extended the ability of this model to investigate how OSA causes cardiac changes. Rats were given transient apnoeas for two durations, 13 and 23 sec., which we termed ‘moderate’ and ‘severe’, respectively. Both groups received 60 apnoeas per hour, 8 hrs per day. Figure 2 shows examples of oxygen saturation (SpO_2_) levels during 13 and 23 sec. apnoeas. While the minimum SpO_2_ ranged from 55.5 to 80.9%, the average minimum oxygen saturation was 70 ± 1.9% and 69 ± 1.8% for moderate (13 sec.) and severe (23 sec.) apnoea groups, respectively (*P* = 0.16). Normoxic SpO₂ was not different for 13 and 23 sec. apnoea groups (96 ± 0.43% and 96 ± 0.03%, respectively (*P* = 0.81)). Apneic episodes were associated with a decreased heart rate in both groups. The 13 sec. apnoea group had a minimum heart rate of 352 ± 11.2 beats per minute, while the 23 sec. apnoea group was 295 ± 15.4 beats per minute (*P* < 0.01) (Fig. 3). There was no difference in the resting heart rate between 13 and 23 sec. apnoea groups (431 ± 11.7 and 421 ± 19.9, respectively (*P* = 0.16)). The weight of OSA rats was reduced compared with controls (*P* < 0.05) (Table 1). Only the severe apnoea group had increased heart mass relative to body mass, although there was a net decrease in body mass throughout the experiment (*P* < 0.05). We next investigated whether chronic OSA altered ECGs in rats (Fig. 4). Table 2 shows ECG parameters measured in control, 13 and 23 apnoea groups. While the P wave duration was increased in both apnoea groups (*P* < 0.05), there was no difference in P wave amplitude (*P* = 0.82). The QT, QTc and QTcr intervals were unchanged (*P* = 0.7, *P* = 0.76 and *P* = 0.46, respectively), but the T wave amplitude increased in both 13 and 23 sec. apnoea groups (*P* < 0.05).

**Table 1:** Mass development of both apnoea groups was inhibited

	Average pre‐apnoea weight (g)	Average post‐apnoea weight (g)	Heart weight/body weight (g/g)	Heart weight/tibia length (g/mm)
Control (*n* = 10)	285.7 (±11.7)	302.4 (±12.0)	0.0035 (±0.0002)	0.028 (±0.0015)
13 sec. apnoea (*n* = 7)	258.0 (±11.6)	261.0 (±10.2)^a^	0.0031 (±0.0002)	0.021 (±0.0008)^a^
23 sec. apnoea (*n* = 9)	267.6 (±11.1)	232.1 (±8.0)^a^	0.0042 (±0.0001)^a^	0.025 (±0.0009)

Thirteen second apnoeas resulted in decreased heart mass relative to tibia length, while relative heart mass was similar to controls. Twenty three seconds apnoeas caused decreased post‐apnoea body mass and increased relative heart mass, although heart weight to tibia length ratio remained unchanged.

a *P* < 0.05 *versus* control.

**Table 2:** ECG parameters in control and chronic obstructive sleep apnoea rats after 2 weeks of apnoea

	Control (*n* = 15)	13 sec. apnoea (*n* = 10)	23 sec. apnoea (*n* = 8)
RR interval (sec.)	0.181 ± 0.004	0.171 ± 0.005	0.170 ± 0.004
Heart rate (BPM)	333.4 ± 6.14	354.8 ± 10.6	354.6 ± 9.9
PR interval (sec.)	0.0488 ± 0.0007	0.0473 ± 0.001	0.0489 ± 0.001
P duration (sec.)	0.0156 ± 0.0002	0.0177 ± 0.0009*	0.0182 ± 0.001**
P amplitude (V)	0.123 ± 0.011	0.117 ± 0.015	0.121 ± 0.01
QRS duration (sec.)	0.0158 ± 0.0007	0.0150 ± 0.0003	0.0151 ± 0.0004
QT interval (sec.)	0.0705 ± 0.004	0.0738 ± 0.003	0.0722 ± 0.004
QTc (sec.)	0.167 ± 0.009	0.158 ± 0.0177	0.176 ± 0.0112
QTcr (sec.)	0.0645 ± 0.004	0.070 ± 0.004	0.0681 ± 0.004
T amplitude (V)	0.127 ± 0.0125	0.173 ± 0.016*	0.198 ± 0.019*

*P < 0.05 *versus* control and **P < 0.01 *versus* control.

### Dysregulated atrial proteins in rats with severe apnoea

OSA patients have high prevalence of atrial fibrillation 21. OSA rats showed increase in the P wave duration, which is suggestive of atrial electrical remodelling 22. Thus, we investigated the molecular changes in the atria caused by chronic OSA. Mass spectrometry has been used in analysis of a variety of compounds such as proteins (proteomics 14, 23, 24), protein–protein interactions (interactomics 15, 25), post‐translational modifications (translatomics 26), or peptides (peptidomics 27). In addition, comparative proteomics analysis of proteins from two or more conditions has been successfully used for unbiased interrogation of biological fluids or cellular lysates 23, 28, 29, 30, 31, 32, 33 for identification of a protein signature characteristic to one condition (*i.e*. cancer) compared with that of controls. When cellular lysates are investigated by proteomics, not only the dysregulated proteins may be identified, but also the cellular pathways which are affected 29, 34. The first indication that a particular metabolic pathway is dysregulated is the levels of the enzymes implicated in the pathway. Therefore, we examined proteomic changes that can be observed in the atria from rats with apnoea compared with controls. Analysing the same amount of protein from each control, moderate apnoea and severe apnoea allowed us to directly compare the relative amounts of particular proteins by directly comparing the spectral counts for each peptide (and then protein). As such, the relative protein levels were compared between the controls and moderate apnoea to find protein indicators for the onset of apnoea, but also proteins that are indicators for advanced stages of apnoea. The comparison between the controls and severe apnoea can also identify protein indicators of the cellular pathways affected by the pathology of the atria due to OSA. To increase the quality of our data, we conducted these experiments in two biological replicates.

**Figure 5:**
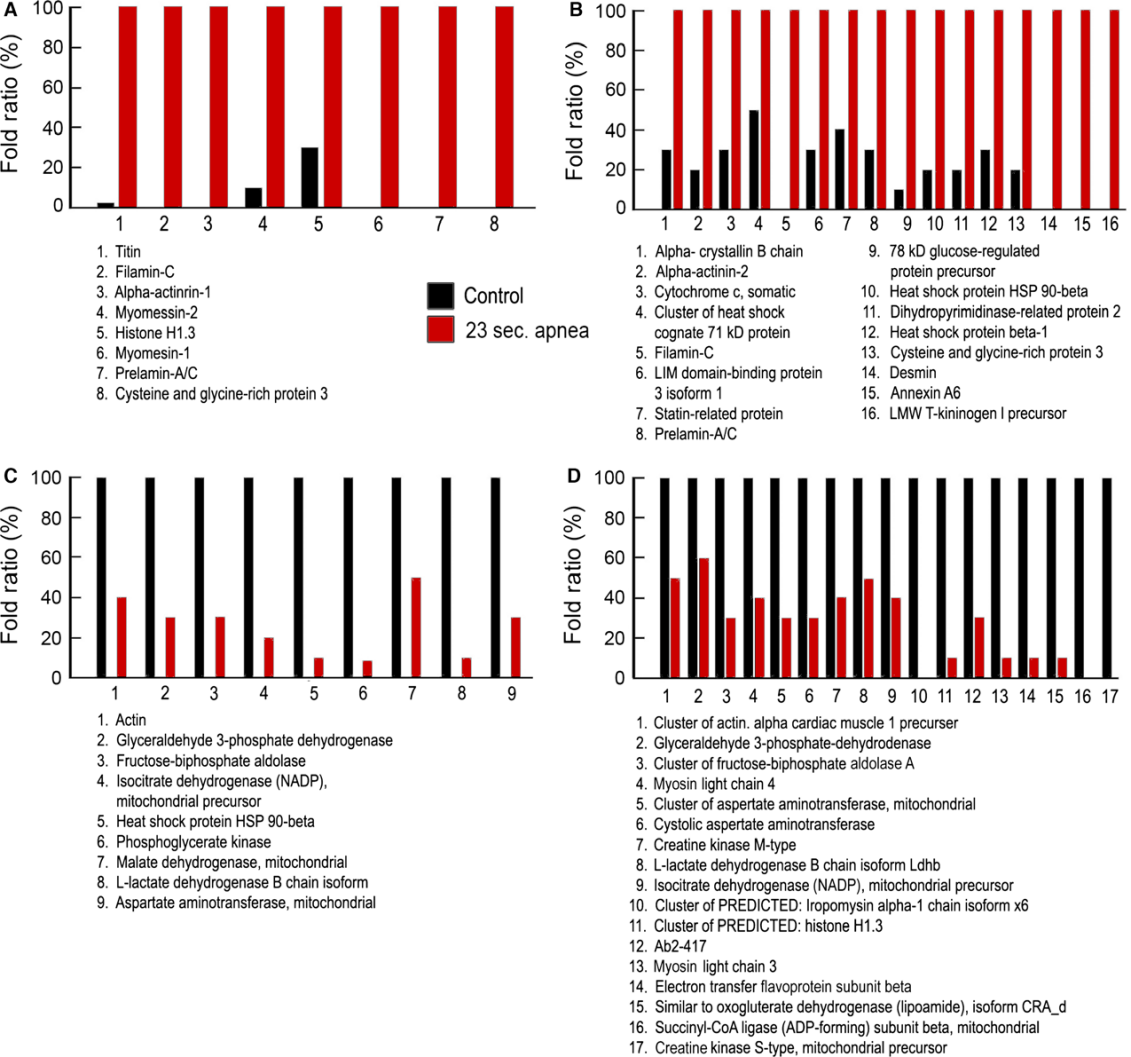
Dysregulated proteins between atria from rats with severe apnoea and healthy controls. (**A**) Up‐regulated proteins in the atria from rats with severe apnoea, as compared with controls in the biological replicate 1. (**B**) Up‐regulated proteins in the atria from rats with severe apnoea, as compared with controls in the biological replicate 2. (**C**) Down‐regulated proteins in the atria from rats with severe apnoea, as compared with controls in the biological replicate 1. (**D**) Down‐regulated proteins in the atria from rats with severe apnoea, as compared with controls in the biological replicate 2. All changes have a *P* < 0.05.

In biological replicate 1, we found a series of proteins that were increased in the atria from rats with severe apnoea (23 sec.), as compared with controls. Among them we found titin (72‐fold), filamin‐C (∞‐fold), alpha‐actinin‐1 (∞‐fold), myomesin‐2 (8.4‐fold), histone H1.3 (3.5‐fold), myomesin‐1 (∞‐fold), prelamin‐A/C (∞‐fold), cysteine and glycine‐rich protein 3 (∞‐fold). The proteins whose level was decreased in severe apnoea, as compared with controls, were actin (0.4‐fold), glyceraldehyde 3‐phosphate dehydrogenase (0.3‐fold), fructose‐bisphosphate aldolase A (0.3‐fold), isocitrate dehydrogenase [NADP], mitochondrial precursor (0.2‐fold), heat‐shock protein HSP 90‐beta (0.1‐fold), phosphoglycerate kinase (0.08‐fold), Malate dehydrogenase, mitochondrial (0.5‐fold), L‐lactate dehydrogenase B chain isoform (0.1‐fold), and aspartate aminotransferase mitochondrial (0.3‐fold). These differences are shown in Figure 5.

In biological replicate 2, we also found a series of proteins that were increased in the atria from rats with severe apnoea, as compared with controls. Among them we found alpha‐crystallin B chain (2.9‐fold), alpha‐actinin‐2 (4‐fold), cytochrome c, somatic (2.9‐fold), cluster of heat‐shock cognate 71 kDa protein (2.3‐fold), filamin‐C (∞‐fold), LIM domain‐binding protein 3 isoform 1(3.3‐fold), statin‐related protein (2.4‐fold), prelamin‐A/C (3.3‐fold), 78 kDa glucose‐regulated protein precursor (8.7‐fold), heat‐shock protein HSP 90‐beta (4.7‐fold), dihydropyrimidinase‐related protein 2 (4.7‐fold), heat‐shock protein beta‐1 (3‐fold), cysteine and glycine‐rich protein 3 (5.4‐fold), desmin (∞‐fold), annexin A6 (∞‐fold), LMW T‐kininogen I precursor (∞‐fold). The proteins whose level was decreased in severe apnoea, as compared with controls, were cluster of actin, alpha cardiac muscle 1 precursor (0.5‐fold), glyceraldehyde 3‐phosphate dehydrogenase (0.6‐fold), cluster of fructose‐bisphosphate aldolase A (0.3‐fold), Myosin light chain 4 (0.4‐fold), cluster of aspartate aminotransferase, mitochondrial (0.3‐fold), cystolic aspartate aminotransferase (0.3‐fold), creatine kinase M‐type (0.4‐fold), L‐lactate dehydrogenase B chain isoform Ldhb (0.5‐fold), isocitrate dehydrogenase [NADP], mitochondrial precursor (0.4‐fold), cluster of PREDICTED: histone H1.3 (0.1‐fold), Ab2‐417 (0.3‐fold), myosin light chain 3 (0.1‐fold), electron transfer flavoprotein subunit beta (0.1‐fold), similar to oxoglutarate dehydrogenase (lipoamide), isoform CRA_d (0.1‐fold), succinyl‐CoA ligase subunit beta, mitochondrial (‐∞‐fold), creatine kinase S‐type, mitochondrial precursor (‐∞‐fold). These dysregulations were seen regardless of which control was used in the comparison. For example, filamin‐C and prelamin‐A/C were always up regulated, and fructose‐bisphosphate aldolase and glyceraldehyde 3‐phosphate dehydrogenase were always down regulated, no matter what controls were used (see Figures S1 and S2).

### Dysregulated atrial proteins in rats with moderate apnoea

**Figure 6:**
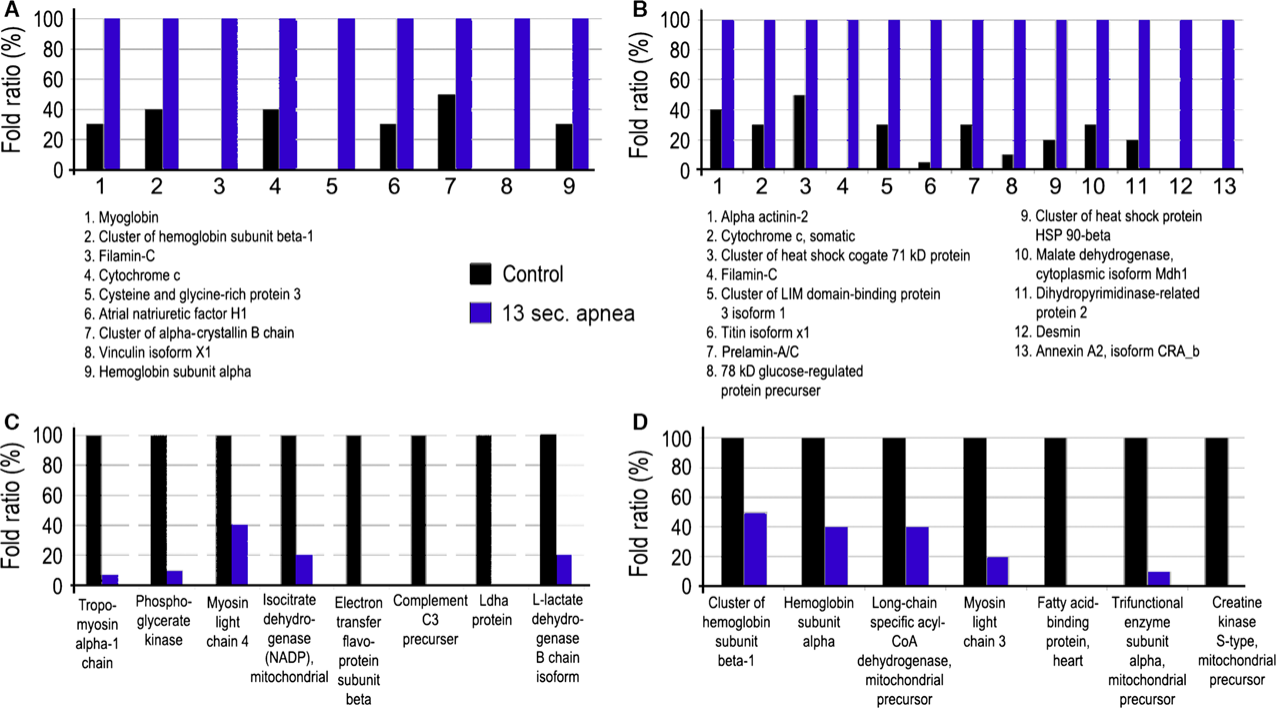
Dysregulated proteins between atria from rats with moderate apnoea and healthy controls. (**A**) Up‐regulated proteins in the atria from rats with moderate apnoea, as compared with controls in the biological replicate 1. (**B**) Up‐regulated proteins in the atria from rats with moderate apnoea, as compared with controls in the biological replicate 2. (**C**) Down‐regulated proteins in the atria from rats with moderate apnoea, as compared with healthy controls in the biological replicate 1. (**D**) Down‐regulated proteins in the atria from rats with moderate apnoea, as compared with controls in the biological replicate 2. All changes have a *P* < 0.05.

In biological replicate 1 (Fig. 6), we found a series of proteins that were increased in the atria from rats with moderate apnoea, as compared with controls. Among them we found myoglobin (3.5‐fold), cluster of haemoglobin subunit beta‐1 (2.6‐fold), cytochrome c (2.3‐fold), cysteine and glycine‐rich protein 3 (∞‐fold), atrial natriuretic factor H1 (3.2‐fold), cluster of alpha‐crystallin B chain (2.2‐fold), vinculin isoform X1 (∞‐fold), hemoglobin subunit alpha (3.3‐fold) and filamin‐C (∞‐fold). The proteins whose level was decreased in moderate apnoea, as compared with controls, were tropomyosin alpha‐1 chain (0.07‐fold), phosphoglycerate kinase (0.1‐fold), myosin light chain 4 (0.4‐fold), isocitrate dehydrogenase [NADP], mitochondrial precursor (0.2‐fold), electron transfer flavo‐protein subunit beta (‐∞‐fold), complement C3 precursor (‐∞‐fold), L‐lactate dehydrogenase B chain isoform Ldhb (0.2‐fold), Ldha (‐∞‐fold).

In biological replicate 2, we also found a series of proteins that were increased in the atria from rats with moderate apnoea, as compared with controls. Among them we found alpha‐actinin‐2 (2.4‐fold), cytochrome c, somatic (3.4‐fold), cluster of heat‐shock cognate 71 kDa protein (2.0‐fold), filamin‐C(∞‐fold), cluster of LIM domain‐binding protein 3 isoform 1 (3.2‐fold), titin isoform X1 (19‐fold), prelamin‐A/C (3.7‐fold), 78 kDa glucose‐regulated protein precursor (6.9‐fold), cluster of heat‐shock protein HSP 90‐beta (5.3‐fold), malate dehydrogenase, cytoplasmic isoform Mdh1 (2.9‐fold), dihydropyrimidinase‐related protein 2 (6.4‐fold), desmin (∞‐fold), annexin A2, isoform CRA_b (∞‐fold). The proteins whose level was decreased in moderate apnoea, as compared with healthy controls, were cluster of hemoglobin subunit beta‐1 (0.5‐fold), hemoglobin subunit alpha (0.4‐fold), long‐chain specific acyl‐CoA dehydrogenase mitochondrial precursor (0.4‐fold), myosin light chain 3 (0.2‐fold), fatty acid‐binding protein, heart (‐∞‐fold), trifunctional enzyme subunit alpha, mitochondrial precursor (0.1‐fold), creatine kinase S‐type, mitochondrial precursor (‐∞‐fold).

### Comparison between severe and moderate apnoea groups

**Figure 7:**
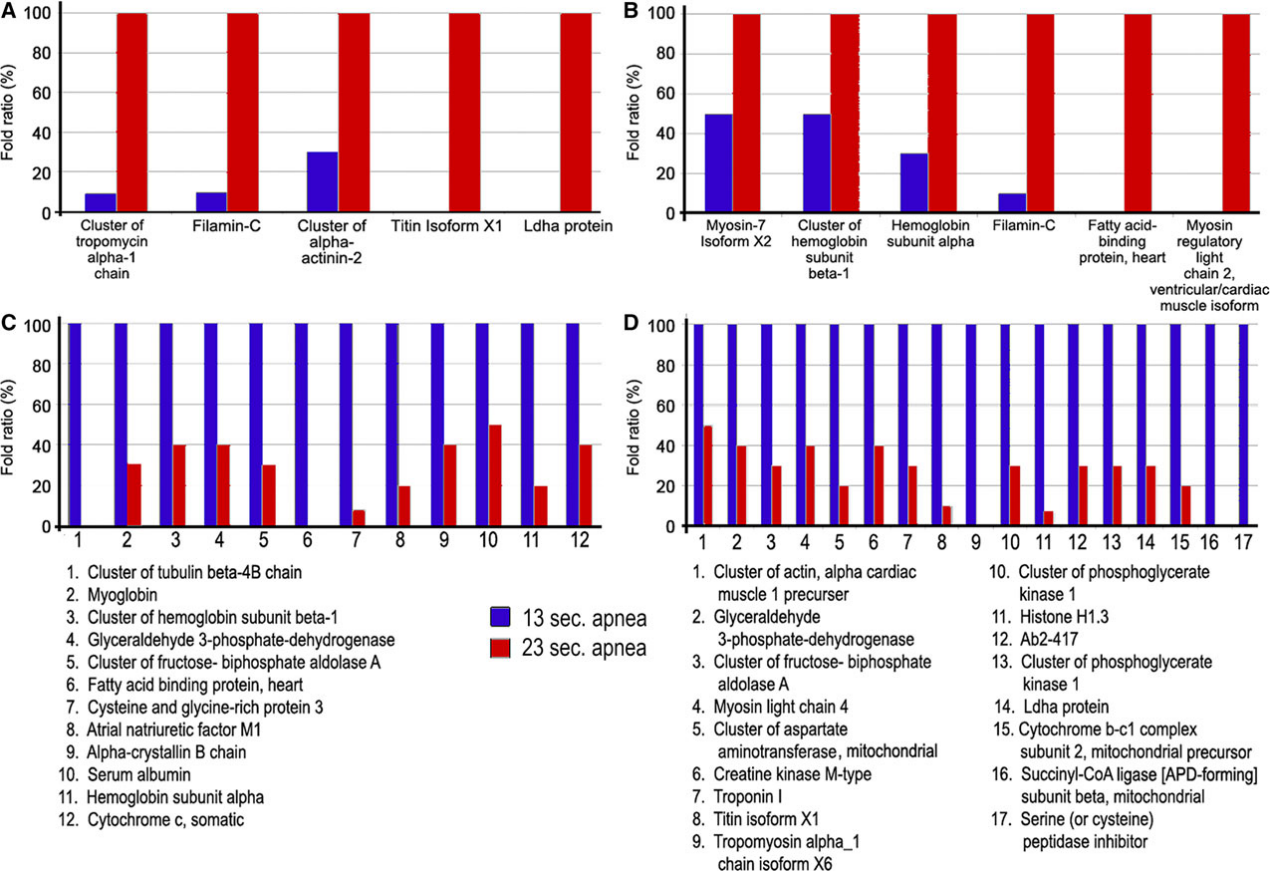
Dysregulated proteins between atria from rats with severe apnoea and moderate apnoea. (**A**) Up‐regulated proteins in the atria from rats with severe apnoea, as compared with moderate apnoea in the biological replicate 1. (**B**) Up‐regulated proteins in the atria from rats with severe apnoea, as compared with moderate apnoea in the biological replicate 2. (**C**) Down‐regulated proteins in the atria from rats with severe apnoea, as compared with moderate apnoea in the biological replicate 1. (**D**) Down‐regulated proteins in the atria from rats with severe apnoea, as compared with moderate apnoea in the biological replicate 2. All changes have a *P* < 0.05.

In biological replicate 1 (Fig. 7), we found a series of proteins that were increased in the atria from rats with severe apnoea, as compared with moderate apnoea. Among them we found cluster of tropomyosin alpha‐1 chain (11‐fold), filamin‐C (7.4‐fold), cluster of alpha‐actinin‐2 (3.8‐fold), titin isoform X1 (**∞**‐fold) and Ldha protein (∞‐fold). The proteins whose level was decreased with severe apnoea, as compared with moderate apnoea, were cluster of tubulin beta‐4B chain (‐∞‐fold), fatty acid binding protein, heart (‐∞‐fold), myoglobin (0.3‐fold), cluster of haemoglobin subunit beta‐1 (0.4‐fold), glyceraldehyde 3‐phosphate dehydrogenase (0.4‐fold), cluster of fructose‐bisphosphate aldolase A (0.3‐fold), cytochrome c, somatic (0.4‐fold), cysteine and glycine‐rich protein 3 (0.08‐fold), atrial natriuretic factor H1 (0.2‐fold), alpha‐crystallin B chain (0.4‐fold), serum albumin (0.5‐fold), hemoglobin subunit alpha (0.2‐fold).

In the biological replicate 2, we found a series of proteins that were increased in the atria from rats with severe apnoea, as compared with moderate apnoea. Among them we found myosin‐7 isoform X2 (2.1‐fold), cluster of haemoglobin subunit beta‐1 (2.2‐fold), hemoglobin subunit alpha (2.9‐fold), filamin‐C (7.3‐fold), fatty acid‐binding protein, heart (∞‐fold), myosin regulatory light chain 2 and ventricular/cardiac muscle isoform (∞‐fold). The proteins whose level was decreased with severe apnoea, as compared with moderate apnoea were cluster of actin, alpha cardiac muscle 1 precursor (0.5‐fold), glyceraldehyde 3‐phosphate dehydrogenase (0.4‐fold), cluster of fructose‐bisphosphate aldolase A (0.3‐fold), myosin light chain 4 (0.4‐fold), cluster of aspartate aminotransferase, mitochondrial (0.2‐fold), creatine kinase M‐type (0.4‐fold), troponin I (0.3‐fold), titin isoform X1 (0.1‐fold), cluster of phosphoglycerate mutase 2 (0.3‐fold), histone H1.3 (0.07‐fold), Ab2‐417 (0.3‐fold), cluster of phosphoglycerate kinase 1 (0.3‐fold), Ldha protein (0.3‐fold), cytochrome b‐c1 complex subunit 2, mitochondrial precursor (0.2‐fold), tropomyosin alpha 1 (‐fold), succinyl‐CoA ligase subunit beta, mitochondrial (‐fold), serine/cysteine peptidase inhibitor (‐fold).

## Discussion

### Pathophysiological characteristics of chronic OSA

Obstructive sleep apnoea patients are susceptible to atrial remodelling and arrhythmias 21, but the mechanisms are unclear. While multiple animal models of OSA exist 8, 35, they do not all recapitulate two key pathophysiological characteristics of OSA, hypoxia and negative intrathoracic pressure. We implemented a recently developed OSA model whereby the apnoeas are produced by inflations of a balloon in the trachea of rats which cause transient obstructions 10, 11 as well as producing hypoxias and negative intrathoracic pressure swings. We extended the ability of this model to produce transient apnoeas for 13 and 23 sec. for 2 weeks. These two apnoea durations are within the range of those experienced by OSA patients 1, 2, and apnoeas last 10 sec. or longer in adults, The 60 apnoeas per hour corresponded to an AHI of 60, which is considered clinically to be severe OSA 1, 2. Throughout the 2 weeks, rats in both groups consistently had apnoeas and transient hypoxias. OSA rats also showed no increase (13 sec. apnoea group) or a decrease (23 sec. apnoea group) in weight compared with controls. It is unclear whether the differences in body weight are the result of fat or muscle loss, and this will need to be assessed in another study.

Obstructive sleep apnoea patients exhibit cardiac electrophysiological changes 36, 37, 38, 39, and we also found that P wave durations and T wave amplitudes were increased after 2 weeks of chronic OSA. For comparison, Baranchuk *et al*. 39 showed that the longer signal averaged P wave durations seen in OSA patients could be decreased with continuous positive airway pressure. In addition, increased P wave durations are also a predictor for the development of atrial fibrillation 22. To our knowledge, this is the first time this OSA model has shown a cardiac electrophysiological phenotype that is also seen in the clinic.

### Effects of OSA on the heart metabolic enzymes

After observing the physiological changes in our OSA rats, we wanted to investigate the extent of molecular remodelling by examining the atrial proteome. We observed that in both biological replicates, 1 and 2, the common glycolytic pathway is dysregulated. Three of the nine enzymes from the glycolytic pathway (fructose‐bisphosphate aldolase A (0.3‐fold), glyceraldehyde 3‐phosphate dehydrogenase (0.3‐fold) and phosphoglycerate kinase (0.08‐fold) were down regulated in the long apnoea group. In addition, two of these three enzymes are main producers of cellular energy: phosphoglycerate kinase produces ATP and most importantly glyceraldehyde 3‐phosphate dehydrogenase produces reducing equivalents (*i.e*. NADH). Only about 5% of ATP in the heart is generated by glycolysis 40. The heart uses fatty acids as a primary energy source, and fatty acid metabolism has also been shown to be down regulated in compensated and uncompensated hypertrophic cardiomyopathies as well as dilated cardiomyopathy 41. Without measuring metabolism directly it is unclear if fatty acid metabolism would compensate for energy deficits caused by a decrease in glycolysis. We found that long‐chain specific acyl‐CoA dehydrogenase (0.4‐fold), which is involved in fatty acid metabolism, was down regulated in moderate apnoea. This suggests that lipid metabolism could be down regulated, in addition to glycolysis, to cause further impairments in energy metabolism.

We also observed that some of the Krebs cycle enzymes are dysregulated: isocitrate dehydrogenase (0.2‐fold) and malate dehydrogenase, mitochondrial (0.5‐fold), both NADH‐producing enzymes are also dysregulated. If the NADH production is dysregulated, the electron transport chain should also be impaired 40. Importantly, changes in cardiac metabolism have been reported in heart failure, including defects in oxidative phosphorylation and dysregulation of mitochondrial proteins 41. Kato *et al*. 42 showed that Dahl salt‐sensitive rats, who were fed a high‐salt diet to induce cardiac disease, had decreases in genes involved in both glycolysis and mitochondrial function during heart failure. We also found a mitochondrial protein involved in electron transport chain down regulated (electron transfer flavoprotein subunit beta (0.1‐fold)). If the aerobic glycolysis is inhibited, one may expect an up‐regulation of the anaerobic glycolysis. However, the first enzyme involved in anaerobic glycolysis, L‐lactate dehydrogenase B chain isoform (0.1‐fold), was found down regulated. Therefore, our data suggest that the entire glycolysis (aerobic and anaerobic) and oxidative capability are down regulated, thus depleting the heart from both energy (ATP) and reducing equivalents (NADH).

### Hypertrophic, structural and maintenance proteins changed with OSA

Other proteins that we found dysregulated were directly involved in muscle contraction and normal cardiac maintenance. For example, titin (72‐fold), filamin‐C (∞‐fold), alpha‐actinin‐1 (∞‐fold), myomesin‐2 (8.4‐fold) and myomesin‐1 (∞‐fold). Filamin‐C is a muscle specific protein which shows the mechanical relationship between the extracellular matrix, the plasma membrane and the actin skeleton. Filamin‐C is essential in maintaining cardiac muscle cell alignment and structure by localizing to intercalated discs of cardiomyocytes. Mutations in filamin‐C are found among patients that have myofibrillar myopathy and cardiac abnormalities 43. Alpha‐actinins are highly conserved actin‐binding proteins. Alpha‐actinin‐1 is a major actin crosslinking protein and non‐muscle isoform in focal adhesions and stress fibres. In muscle cells, myomesin stabilizes the three‐dimensional conformation of the thick filaments. Myomesin‐1 and myomesin‐2 are proteins that comprise the MYOM family located in the M band. Myomesin is a cross linker of myosin filaments, and the C‐terminal end of the titin extends into the M line, where it binds tightly to myomesin‐1 and myomesin‐2 44. Other up‐regulated proteins such as prelamin‐A/C (∞‐fold) or cysteine and glycine‐rich protein 3 (∞‐fold) are also involved in normal cardiac maintenance and are up regulated in response to chronic severe 23 sec. apnoeas. For example, prelamin‐A/C helps to maintain the volume and strength of skeletal muscle 45 and when its gene is mutated, it directly induces severe aortic stenosis and hypertrophic cardiomyopathy, along with atypical fat distribution and insulin resistance 46. The cysteine and glycine‐rich protein 3, also known as muscle LIM protein, can be found at the Z‐disc of cardiac muscle 47, can translocate to the nucleus and is implicated in mecho‐sensory actions 48. Importantly, partial deletion of muscle LIM protein attenuated pro‐hypertrophic the calcineurin–nuclear factor of activated T cells (NFAT) signalling pathway 49.

Not surprisingly, the effect of moderate apnoea on the heart partly resembles that of severe. Proteins like filamin‐C (∞‐fold), titin isoform X1 (19‐fold), filamin‐C(∞‐fold), prelamin‐A/C (3.7‐fold), alpha‐actinin‐2 (2.4‐fold) are up regulated, as in severe 23 sec. apnoea. Other proteins like myoglobin (3.5‐fold), cluster of haemoglobin subunit beta‐1 (2.6‐fold), hemoglobin subunit alpha (3.3‐fold) suggests a lower supply of oxygen, compensated by up‐regulation of these proteins in moderate apnoea. Proteins like cluster of heat‐shock cognate 71 kDa protein (2.0), 78 kDa glucose‐regulated protein precursor (6.9‐fold), cluster of heat‐shock protein HSP 90‐beta (5.3‐fold) and annexin A2, isoform CRA_b (∞‐fold) are involved in heat‐stress response 23, 50, 51.

Atrial natriuretic factor H1 (ANP) was increased (3.2‐fold) in moderate apnoea. ANP is elevated by atrial distension 40 and plays a role in volume homoeostasis 40, 52. ANP has also been shown to enhance lipolysis 53. Certain OSA patient populations also have increased lipolysis 54. In our study, significant weight differences were also observed when comparing 13 and 23 sec. apnoea groups, indicating decreased rate of mass development as apnoea severity increases, although weight data have not been compared with the amount of adipose tissue in any of the OSA groups. On the other hand, ANP is decreased in severe apnoea when compared with moderate. This suggests that other factors are playing a role in OSA‐induced weight loss.

Heat‐shock proteins were also changed with OSA. Heat‐shock protein 70 (HSP70) and heat‐shock protein 90 (HSP90) are families of protective chaperones in the transport of malformed proteins and prevent aggregation of proteins affected by cytotoxic stressors such as heat and oxidation. Chronic intermittent hypoxia may increase the concentration of oxidative species, thus up‐regulating heat‐shock proteins 55. HSP90 plays an important role in the transport of glucocorticoids, binding to the glucocorticoid receptor, causing it to maintain a conformation that is able to bind with glucocorticoids such as cortisol 56. One variant of the HSP70 family, glucose‐regulated protein 78 (GRP78), is responsible for glycosylation and assembly of transmembrane proteins. GRP78 is down regulated as glucose concentration decreases and as available glucose nears depletion, GRP78 mRNA concentration increases rapidly 57. It is possible that down‐regulation of proteins involved in glycolysis contributes to alternations in production of GRP78.

Proteins that are thought to induce cardiomyopathy were also up regulated. Examples of such proteins are cysteine and glycine‐rich protein 3 (∞‐fold) (or muscle LIM protein, as described above) and cluster of alpha‐crystallin B chain (2.2‐fold) 58, 59, 60, 61, 62. Other proteins like phosphoglycerate kinase (0.1‐fold), L‐lactate dehydrogenase B chain isoform Ldhb (0.2‐fold) and isocitrate dehydrogenase [NADP], mitochondrial precursor (0.2‐fold) were down regulated in moderate apnoea, further supporting the idea that, as with longer apnoea, dysregulation of glycolysis indeed may happen in short apnoea, although of a lower intensity. This further suggests that dysregulation of glycolysis actually starts in moderate apnoea and worsens in severe apnoea.

### Protein changes between moderate and severe apnoea groups

Many proteins found up regulated in moderate apnoea are also up regulated in severe apnoea. For example, Myosin‐7 isoform X2 (2.1‐fold), cluster of haemoglobin subunit beta‐1 (2.2‐fold), hemoglobin subunit alpha (2.9‐fold), filamin‐C (7.3‐fold), were up regulated in severe, compared with moderate apnoea. Furthermore, the enzymes involved in aerobic and anaerobic glycolysis and in the electron transport chain are down regulated in severe apnoea, as compared to moderate apnoea: glyceraldehyde 3‐phosphate dehydrogenase (0.4‐fold), Cluster of fructose‐bisphosphate aldolase A (0.3‐fold), phosphoglycerate mutase 2 (0.3‐fold), phosphoglycerate kinase 1 (0.3‐fold), Ldha protein (0.3‐fold), cytochrome b‐c1 complex subunit 2, mitochondrial precursor (0.2‐fold). Cytochrome b‐c1 complex subunit 2 is a protein used in the mitochondrial respiratory chain to transfer electrons from the ubiquinol to cytochrome C 63. These data suggest that apnoea, regardless of whether it is moderate or severe, has the same effect on the heart, that is, a decrease in glycolysis and energy producing capacity as well as a compensatory cardiac hypertrophy.

### Limitations

Despite our novel and compelling findings, the study had limitations. First, we did not directly measure metabolic processes directly, and therefore, we cannot fully assess the physiological impact the dysregulation of glycolytic and mitochondrial proteins. Second, OSA is a progressive and chronic disease. While our study assesses two weeks of chronic OSA, longer study times may produce greater cardiac remodelling and lead to further pathology or compensatory adaptations.

## Conclusions

In conclusion, we show that this recently developed model can be extended to study the cardiac changes induced by OSA. In addition to electrocardiographic changes, our experiments showed that OSA induces severe protein dysregulations, suggesting impairment of energy metabolism. OSA also induced up‐regulation of sarcomeric and pro‐hypertrophic proteins. These results will help elucidate the complex pattern of changes occurring with OSA which further leads to cardiac disease.

## Conflict of interest

The authors confirm that there are no conflict of interests.

## Supporting information


**Figure S1** Comparison of up‐regulated proteins between two biological replicates (1 and 2).
**Figure S2** Comparison of down‐regulated proteins between two biological replicates (1 and 2).
