# Supplementary material for: Atrial electrophysiological and molecular remodelling induced by obstructive sleep apnoea
Source: J Cell Mol Med. 2017 Apr 12;21(9):2223–35. doi: 10.1111/jcmm.13145 (PMC5571519; doi:10.1111/jcmm.13145)
Supplement: Supplementary file 1 — Figure S1 Comparison of up‐regulated proteins between two biological replicates (1 and 2). Figure S2 Comparison of down‐regulated proteins between two biological replicates (1 and 2). [file JCMM-21-2223-s001.docx]

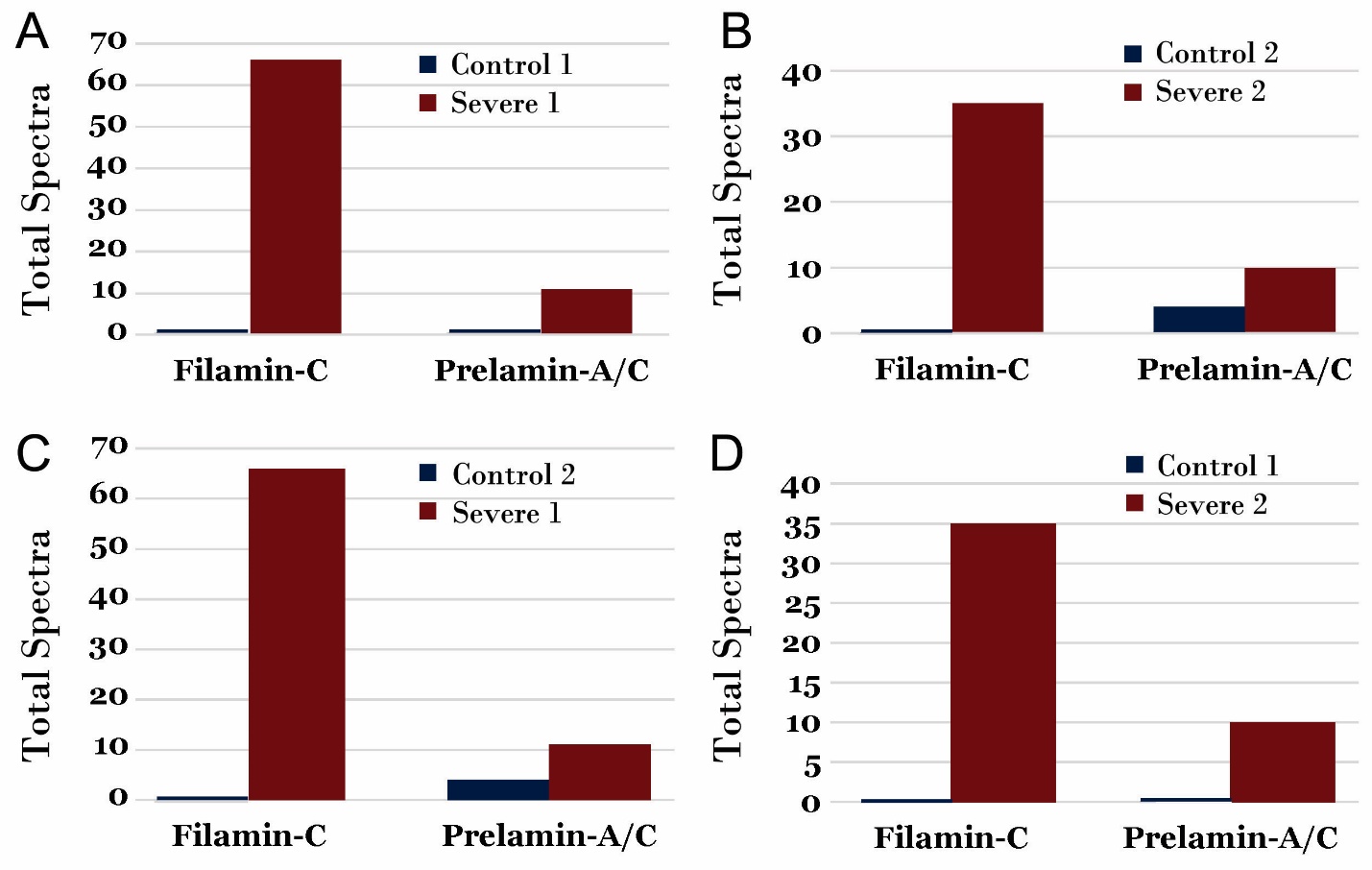


**Supplemental Figure 1. Comparison of up-regulated proteins between two biological replicates (1 and 2).**


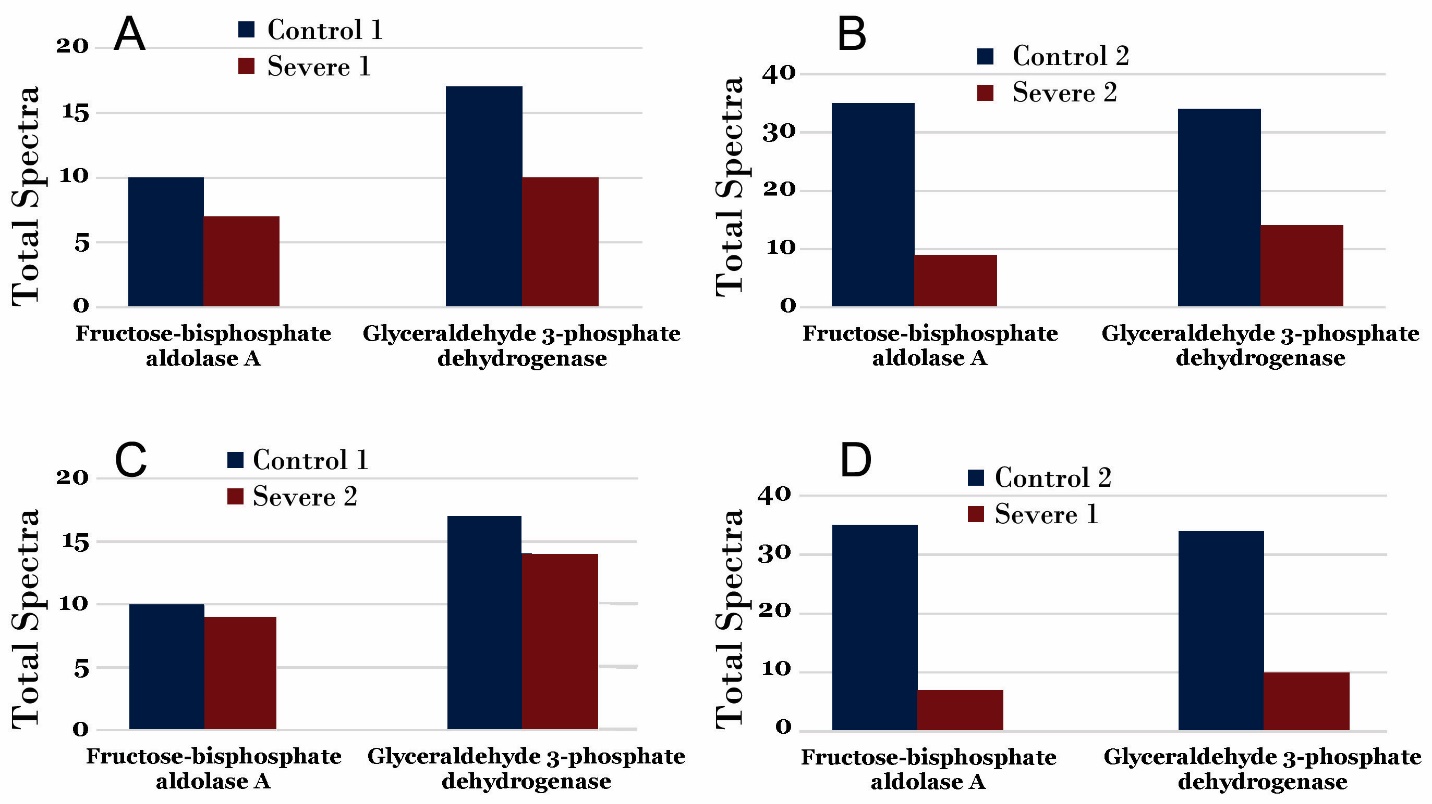


**Supplemental Figure 2. Comparison of down-regulated proteins between two biological replicates (1 and 2).**
